# Marine Omega-3 Fatty Acids, Complications of Pregnancy and Maternal Risk Factors for Offspring Cardio-Metabolic Disease

**DOI:** 10.3390/md16050138

**Published:** 2018-04-24

**Authors:** Melinda Phang, Michael R. Skilton

**Affiliations:** Boden Institute of Obesity, Nutrition, Exercise and Eating Disorders, Sydney Medical School, University of Sydney, Camperdown 2006, Australia; melinda.phang@sydney.edu.au

**Keywords:** cardio-metabolic disease, marine omega-3 fatty acids, maternal risk factors

## Abstract

Marine omega-3 polyunsaturated fatty acids (n-3 PUFA) are important nutrients during periods of rapid growth and development in utero and infancy. Maternal health and risk factors play a crucial role in birth outcomes and subsequently offspring cardio-metabolic health. Evidence from observational studies and randomized trials have suggested a potential association of maternal intake of marine n-3 PUFAs during pregnancy with pregnancy and birth outcomes. However, there is inconsistency in the literature on whether marine n-3 PUFA supplementation during pregnancy can prevent maternal complications of pregnancy. This narrative literature review summarizes recent evidence on observational and clinical trials of marine n-3 PUFA intake on maternal risk factors and effects on offspring cardio-metabolic health. The current evidence generally does not support a role of maternal n-3 PUFA supplementation in altering the incidence of gestational diabetes, pregnancy-induced hypertension, or pre-eclampsia. It may be that benefits from marine n-3 PUFA supplementation are more pronounced in high-risk populations, such as women with a history of complications of pregnancy, or women with low marine n-3 PUFA intake. Discrepancies between studies may be related to differences in study design, dosage, fatty acid interplay, and length of treatment. Further prospective double-blind studies are needed to clarify the impact of long-chain marine n-3 PUFAs on risk factors for cardio-metabolic disease in the offspring.

## 1. Introduction

The association between early life insults in utero with an increased risk of developing several non-communicable diseases in later life has been well established [1]. Early life risk factors that may predispose the offspring to cardio-metabolic disease in later life include impaired fetal growth, preterm birth, gestational diabetes, pre-eclampsia, and maternal obesity [2]. The health and nutritional status of the mother during pregnancy is also likely to be a key factor in healthy prenatal development and programming of adult disease [3]. Examples of extreme maternal undernutrition, such as the Dutch Hunger Winter [4] and Biafran famine [5], have shown that severe nutritional exposures in critical periods of pregnancy, whether in healthy or high-risk mothers, have the potential to influence offspring cardio-metabolic health [6]. More subtle differences in dietary intake and nutrition are of greater relevance to people living in developed nations, with secure food supply. Several studies have now established that the quantity and quality of maternal dietary fat intake have profound health implications during and after pregnancy [7]. In this respect, polyunsaturated fatty acids (PUFAs), particularly the marine omega-3 (n-3) PUFAs, play a critical role.

The most biologically active n-3 PUFAs are eicosapentaenoic acid (EPA) and docosahexaenoic acid (DHA). These marine n-3 PUFAs have been shown to have multiple beneficial effects, including improving childhood development when ingested during pregnancy [8]. EPA and DHA along with the omega-6 (n-6) fatty acid—arachidonic acid (AA) are essential structural components in cellular membranes. While consumption of n-6 PUFAs are also associated with a lower risk of premature mortality when compared to saturated fats [9], they and n-3 PUFAs have opposing effects on key inflammatory pathways. Specifically, both EPA and AA compete for the same cyclooxygenase enzyme to serve as precursors for biologically active eicosanoids. Diets that are rich in the n-6 PUFAs produce potent proinflammatory eicosanoids, whereas a diet with a more balanced intake of n-6 and n-3 PUFAs produce less inflammatory and less immunosuppressive eicosanoids [10], all which may play a role in maternal and fetal health. 

We have previously described the role of n-3 PUFAs as moderators and mediators of the association of impaired fetal growth with later atherosclerotic and hemodynamic disease [11]. This current narrative review describes the current evidence from observational and clinical trials, including systematic reviews on the effects of maternal marine n-3 PUFA intake on key maternal risk factors, specifically preterm birth, pre-eclampsia, gestational diabetes, maternal obesity, and breastfeeding, all of which are involved in the programming of offspring cardio-metabolic disease.

## 2. Results

### 2.1. Duration of Gestation

There is some evidence that birth weight and length of gestation are increased among communities with a high habitual fish intake attributed to the n-3 PUFA content of marine foods [12,13]. It has been postulated that n-3 PUFAs may increase fetal growth rate by increasing the ratio of biologically active prostacyclins to thromboxanes, reducing blood viscosity, and thereby facilitating placental blood flow [14]. Alternatively they may prolong gestation by inhibiting the production of the proinflammatory n-6 AA-derived prostaglandins that induce cervical ripening [15], thus delaying the initiation of labour [16]. The fetoplacental unit is supplied with long chain polyunsaturated fatty acids (LC-PUFA) from the maternal circulation, which is influenced by maternal LC-PUFA intake and endogenous synthesis. The proinflammatory prostaglandins, derived from n-6 AA within the uteroplacental unit in normal pregnancy, are countered by a local production of prostaglandins derived from n-3 PUFA. Therefore, optimising n-3 PUFA intake in pregnancy, in relation to n-6 AA levels, may mechanistically lead to lower risk of preterm birth. Improving the n-3 status in pregnancy has emerged as a promising strategy to prevent preterm birth and may have value as a prophylactic intervention in some women, particularly those with high-risk pregnancies. Indeed, cohort studies have supported the beneficial effects of n-3 PUFAs in this regard, with evidence for a dose-dependent relationship between marine food intake in pregnancy and infant birth weight [17], as well as a reported association between low consumption of marine foods in early pregnancy with preterm delivery and low birth weight [18]. In a randomized controlled trial of 533 pregnant women, supplementation of 2.7 g/day of n-3 PUFA commenced at the 30th week of gestation was associated with longer gestation [19]; this affected the maternal thromboxane and prostacyclin production [20] and increased the concentrations of n-3 PUFAs in umbilical blood and tissues [21]. Pregnancies in the n-3 PUFA group were on average 4.0 (95% CI 1.5, 6.4) days longer than those in the olive-oil group; the difference in birth weight was 107 g (95% CI 1, 214) [19]. The single largest randomized controlled trial of n-3 PUFA supplementation during pregnancy (DHA to Optimize Mother and Infant Outcome (DOMInO)), and the most recent Cochrane systematic review of marine oil supplementation in pregnancy both support n-3 PUFA supplementation during pregnancy, increasing the mean duration of gestation by 2 days and producing a 40–50% reduction in early preterm birth [22,23]. In the Cochrane review, two of the six included trials reported that women of high-risk pregnancies allocated to receive n-3 DHA-rich fish oil had a lower risk of early preterm birth compared with the control group [22]. While the DOMInO trial also reported fewer preterm births in the DHA group compared to the control group (1.09% vs. 2.25%; adjusted RR, 0.49 (95% CI: 0.25, −0.94); *p* = 0.03), there was also an increase in the incidence of post-term inductions or post -term prelabor caesarean section in the DHA group compared with control (17.6% vs. 13.7%, adjusted RR 1.28 (95% CI: 1.06 to 1.54); *p* = 0.01). The DHA supplemented group also had fewer infants that were of low birth weight (3.41% vs. 5.27%; adjusted RR, 0.65 (95% CI: 0.44–0.96); *p* = 0.03), and higher mean birth weight (mean difference: 68 g (95% CI: 23–114 g); *p* = 0.003) [23]. However, mean birth weight z scores (corrected for gestational age and sex) did not differ between groups, consistent with group differences in birth size being largely a function of differences in gestational age at birth. Two recent systematic reviews on the effects of n-3 PUFAs on preterm birth also reported higher mean birth weights and gestational age at delivery in women that received marine n-3 PUFAs compared with control [24,25]. In the most recent review [24] which included nine trials, the risk of early preterm delivery (<34 weeks) was reduced by 58% (RR 0.42 (95% CI: 0.27–0.66); *p* = 0.0002) and overall preterm delivery (<37 weeks) by 17% (RR 0.83 (95% CI: 0.70–0.98); *p* = 0.03) with n-3 PUFA supplementation. Interestingly, a large population study of 67,007 women from the Norwegian Mother and Child Cohort Study [26] reported that marine n-3 PUFA supplementation was not significantly associated with preterm delivery, whereas increasing marine food intake was associated with lower prevalence of preterm delivery (adjusted HR 0.76 [95% CI: 0.66, 0.88] for 1–2 servings/week (20–40 g/day), 0.72 [95% CI: 0.62, 0.83] for 2–3 servings/week (40–60 g/day), and 0.72 [95% CI: 0.61, 0.85] for ≥3 servings/week (>60 g/day), *p*-trend < 0.001).

Taken together, this evidence supports marine n-3 PUFA intake during pregnancy causing a subtle, yet clinically meaningful increase in overall gestational duration. Nonetheless, this has not yet been confirmed as a primary outcome in a prospective randomized trial and cannot necessarily be generalized to the population of other high-risk groups. Furthermore, the dose, duration, and commencement of n-3 PUFA supplementation in pregnancy that provides the maximal benefit is not known. Accordingly, there are currently insufficient data to support a recommendation of n-3 PUFA supplementation or increased dietary intake specifically for reducing the risk of preterm birth across the population.

With regard to the effects of postnatal n-3 PUFA supplementation in preterm infants, conflicting results have been reported. Most follow-up studies have largely focused on neurodevelopmental outcomes and growth. Few have reported cardio-metabolic endpoints. In preterm infants (<33 weeks), those that were fed an n-3 PUFA-enriched formula had greater lean mass and lower fat mass at 1 year of corrected age compared to the control group [27]. Conversely, a 10-year follow-up study of a preterm cohort showed no differences in growth or blood pressure between those assigned to unsupplemented or n-3 PUFA supplemented formulas from birth to 9 months post term. When stratified by sex, girls that received the active supplemented formula showed increased weight (42.2 kg vs. 39.9 kg, *p* = 0.05), adiposity and blood pressure (BP) (systolic/diastolic BP, 111/65 vs. 106/61 mm Hg, *p* = 0.04) compared to the control group [28]. There is conflicting evidence regarding growth, both weight and length, in infancy post marine n-3 PUFA supplementation. One trial showed increased weight and length at 2 months post-term in supplemented infants [29], another trial reported no effects on growth [30], while three trials found that n-3 PUFA supplemented infants grow less well than controls [31,32,33]. Infant growth post n-3 PUFA supplementation in the postnatal period and during pregnancy should continue to be assessed as a secondary outcome in clinical trials, although current evidence suggests that there is no meaningful effect.

### 2.2. Pre-Eclampsia and Pregnancy-Induced Hypertension

Pre-eclampsia is a potentially dangerous complication in the second half of pregnancy and is characterized by hypertension, abnormal amounts of protein in the urine, and other systemic disturbances [34]. It is the primary reason for elected preterm birth. There is a bi-modal distribution of fetal growth associated with pre-eclampsia, such that those with early onset pre-eclampsia are at higher risk of delivering small-for-gestational age preterm newborns, and those with late-onset are more likely to deliver appropriate or even large-for-gestational age term newborns [35]. Placental stress appears to a play a key role in the pathophysiology of pre-eclampsia through adverse impacts on placental function and fetal growth [36]. The stress is thought to induce the placenta to release inflammatory cytokines and anti-angiogenic factors, among others, which culminate in a maternal inflammatory response and endothelial dysfunction [37]. Adding anti-oxidants or blocking the stress or inflammatory pathways in vitro attenuates these effects and opens possibilities for therapeutic intervention. A number of studies have thus examined the different nutrients in the maternal diet during pregnancy which might influence risk for pre-eclampsia or gestational hypertension. In a cohort of 1718 women who had their diet assessed in the first trimester, there was some evidence to support a lower risk of pre-eclampsia in those with a higher intake of DHA and EPA (odds ratio 0.84 (95% CI: 0.69–1.03) per 100 mg/day), or fish (OR 0.91 (95% CI: 0.75–1.09) per serving/day). No associations were observed with intakes of calcium, vitamins C, D, or E, milk, magnesium, folate, or with intakes of n-6 or trans fatty acids [38]. Indeed, studies have shown that women with pre-eclampsia have altered levels of LC-PUFA. Reduced maternal erythrocyte DHA levels observed in term pre-eclampsia was proposed as one of the key factors affecting membrane stability contributing to the pathophysiology of pre-eclampsia [39,40]. Increased oxidative stress is another possible major causative factor leading to lower plasma DHA and n-3 PUFA levels in the mothers with pre-eclampsia and preterm pregnancies [39,40].

Studies have suggested potential benefits of dietary marine n-3 PUFA and/or fish oil supplementation, based upon either dietary intake or levels of biomarkers among women who develop pre-eclampsia [41,42,43]. However, not all observational studies and randomized trials have shown beneficial effects of n-3 PUFAs [44,45]. In the latest Cochrane review that included all pregnant women regardless of their risk for pre-eclampsia, there were no differences in the relative risk of high blood pressure during pregnancy (RR 1.09, (95% CI: 0.90 to 1.33); 5 trials, 1831 women) or the incidence of pre-eclampsia (RR 0.86, (95% CI: 0.59 to 1.27); 4 trials, 1683 women), between women who received long-chain n-3 PUFA supplements and their respective control groups [22]. A recent meta-analysis which included 11 randomized controlled trials also showed that long-chain n-3 PUFA supplementation was not associated with reduced risks for pregnancy-induced hypertension (RR = 1.06 (95% CI: 0.89, 1.20); *p* = 0.66) or pre-eclampsia (RR = 0.93 (95% CI: 0.74, 1.16); *p* = 0.51) in either low- and high-risk pregnancies [46].

### 2.3. Maternal Diabetes and Gestational Diabetes

Maternal diabetes, including pre-existing type 2 diabetes, type 2 diabetes first diagnosed during pregnancy, and gestational diabetes, exposes the fetus to an excess of nutrients resulting in fetal over-nutrition. The developmental over-nutrition hypothesis was first proposed in 1954 [47], explaining the relationship between maternal diabetes during pregnancy and fetal overgrowth and increased adiposity. Maternal hyperglycemia is associated with the increased placental transfer of glucose, resulting in fetal hyperglycemia and increased insulin production, with the resultant effect being an increase in insulin-mediated fetal growth, or macrosomia [48]. Infants with macrosomia of mothers with diabetes are prone to glucose intolerance, obesity, and diabetes during childhood and adulthood [49], putatively via programming induced by the fetal environment, in addition to genetic factors and the postnatal environment in which the child is raised. Disturbances not only in the metabolism of carbohydrates but also in lipids, which have been observed in newborns of women with diabetes, may influence the metabolic profile later in life [50]. Evidence from experimental and clinical studies demonstrates the beneficial effects of n-3 PUFA consumption during diabetes on insulin function [51], oxidative stress, and in vivo lipid peroxidation [52,53]. Recently, a Norwegian cohort study of 33,740 women reported that consumption of lean fish (75–100 g/day) was associated with a 30% lower risk of developing type 2 diabetes, compared with women who do not consume fish [54]. Clinical trials conducted in people with type 2 diabetes suggest that n-3 PUFA supplementation has at best marginal effects on metabolic control, despite modest reductions in hypertriglyceridemia [55]. However, controversies exist regarding the potential role of n-3 PUFA in treatment and prevention of diabetes during pregnancy, where promising findings in animal models have been unable to be duplicated in humans [51,56]. A systematic review of the effect of long chain n-3 PUFA supplementation in low-risk pregnancy reported no difference between supplemented and non-supplemented women in the rate of gestational diabetes (RR = 0.73; 95% CI: 0.22, 2.37) [57]. More recently, a large randomized controlled trial of 2399 pregnant women found that DHA supplementation (800 mg/day) during the second half of pregnancy did not reduce the risk of gestational diabetes mellitus or pre-eclampsia in mothers [58].

Regarding metabolic health, animal and human studies have shown that maternal diabetes induces maternal hyperlipidemia, further contributing to macrosomia in the newborn [59,60,61]. Post mortem studies demonstrate increased extent and severity of aortic atherosclerosis in fetuses and children of women with hyperlipidemia [62,63]. In experimental studies in animals, a high EPA diet markedly lowered triglycerides in diabetic dams and their macrosomic offspring [60], as well as reducing the incidence of macrosomia from 64 to 48% [61]. Furthermore, the feeding of a high EPA diet to macrosomic offspring during adulthood reduced serum triglyceride levels in liver, with lower rates of hyperglycemia and hyperinsulinemia than those on unsupplemented chow [60]. Taken together, these studies in animals indicate that n-3 PUFA attenuate hyperlipidemic processes in diabetic pregnant animals and their macrosomic newborns. Whether such beneficial effects exist in humans to correct metabolic anomalies associated with insulin-mediated increases in fetal growth are of interest.

### 2.4. Maternal Obesity

Maternal overweight and obesity have implications on human health, as pregnancy complications such as large-for-gestational age, gestational diabetes, and pre-eclampsia are more prevalent in these women [64]. Maternal obesity and excessive gestational weight gain, resulting in over-nutrition of the fetus, are important contributors to increased offspring birth weight. Adipogenesis and lipogenesis are highly sensitive to the nutritional environment in utero and in early postnatal life, with both under and over nutrition having been shown to play a role in impacting cardiovascular function in infants, children and adults [65]. While maternal obesity is associated with long-term risk of obesity in the offspring, Mendelian randomization studies suggest that such an association is not causal [66].

Obesity is associated with metabolic inflammation, characterized by elevated adipose tissue, systemic pro-inflammatory cytokine levels, and adipose tissue macrophage accumulation [67]. This physiological state of low grade inflammation is furthermore enhanced during pregnancy in women with obesity [68]. Additionally, these changes extend to the placenta, thereby exposing the fetus to an inflammatory environment during development [69]. In the context of obesity, n-3 PUFAs have been documented to exert anti-inflammatory effects by modulating adipose tissue, skeletal muscle, and hepatic function [70]. In pregnant women with overweight or obesity, supplementation with n-3 PUFA (DHA: 1200 mg/day; EPA: 1200 mg/day) from week 16 gestation to delivery lowered the expression of inflammatory cytokines (IL6, IL8, TNFα and TLR4 mRNA) in adipose and placental tissue compared with the control group (*p* < 0.001), as well as decreasing C-reactive protein at time of delivery [71]. In the adipose tissue of obese rats, n-3 PUFA modulates the secretion profile of adipokines and decreases pro-inflammatory cytokine secretion including tumor necrosis factor-α and interleukin-6 [72]. In rats fed a high fat diet, n-3 PUFAs increased fatty acid oxidation and inhibited lipogenesis in the liver, causing fatty acids to be preferentially oxidized rather than being stored [73]. One of the most well described mechanisms for the effects of marine n-3 PUFAs is their competition with n-6 PUFAs for incorporation into the phospholipid membrane. It is plausible that the relative excess of n-6 PUFA and the paucity of marine n-3 PUFA in typical Western diets play a role in adverse pregnancy outcomes in women with obesity. A study using Fat-1 transgenic mice (capable of endogenously converting n-6 PUFA to n-3 PUFA), demonstrated the potential to reduce inflammation associated with diet-induced obesity and improve metabolic outcomes in offspring [74]. Fat-1 mice are protected from adverse effects of a diet rich in n-6 PUFA, including adipose tissue macrophage accumulation and systemic increases in pro-inflammatory cytokines. The adult male offspring from Fat-1 high fat diet mothers displayed less adiposity, hepatic lipid accumulation, adipose tissue macrophages, and insulin resistance, compared with offspring from wild type high fat diet mothers [74]. Despite the strong evidence supporting n-3 PUFA driving improvements in obesity and related insulin resistance in animal models, including in the context of pregnancy, human studies are yet to provide strong support of such findings.

### 2.5. Breastfeeding

The lipids of human milk are of critical importance for the infant as a major energy source to support appropriate growth and development. Approximately half of the calories in human milk are from fat. Elongation and desaturation enzymes for PUFA conversion are present in the fetal liver early in gestation, although their activity appears to be low before birth [75]. Therefore, the n-6 and n-3 LC-PUFA accumulated in the fetus in utero are derived predominantly through placental transfer. Furthermore, n-6 and n-3 LC-PUFA concentrations in cord blood are influenced by the maternal diet [76], suggesting a direct link between maternal dietary intake and fetal n-6 and n-3 bioavailability. In infancy, the conversion rates of linoleic acid (LA) to AA and of alpha-linolenic aid (ALA) to DHA are influenced by genetics, gender, and the amount of precursor fatty acids available in the maternal diet [77].

A growing body of evidence has shown that breastfeeding is associated with long term benefits for the infant, such as a reduced risk of developing obesity during childhood [78]. A systematic review of studies investigating the association between infant feeding and obesity concluded that breastfeeding is associated with a reduced risk of childhood obesity, compared with formula feeding (OR: 0.87; 95% CI: 0.85–0.89) [79]. Several studies have also reported a dose-dependent effect of breastfeeding duration on the prevalence of obesity [80]. It has been hypothesized that LC-PUFA are also involved in the anti-diabetic effects of breastfeeding for the offspring [81], supported by evidence that breast milk fatty acids, including n-3 PUFA, are more readily incorporated into erythrocytes than are those from infant fish oil supplementation [82].

Human milk provides LA, ALA, DHA and AA. While the level of AA is relatively constant, the level of DHA is variable and dependent on maternal diet in a dose dependent fashion [83]. Indeed, supplementing lactating women with 200 mg/day of DHA increases breast milk DHA levels almost two-fold, compared with unsupplemented controls (0.37 vs. 0.21 wt %, *p* = 0.003) [84]. For the infant, a threshold exists whereby consumption of human milk DHA levels above 0.8% of total fatty acids does little to increase the plasma or erythrocyte DHA content, although below this threshold there appears to be a dose–tissue response [85]. Interestingly, on average women who are classified with overweight or obesity before pregnancy tend to breastfeed for shorter durations than healthy weight women [86]. Longer durations of breastfeeding are consistently associated with infants who are lighter and shorter at 1 year of age than are infants who have shorter durations of breastfeeding or were not breastfed at all [87,88]. However this does not appear to relate to n-3 LC-PUFA levels, as evidenced by trials administrating 2–3 g/day of (n-3) LC-PUFA during pregnancy or lactation having no effects on growth in healthy term infants up to ~2 years of age [85,89,90]. In one randomized controlled trial, fish oil supplementation in lactating mothers did not affect the weight or length gain of the infants during the first year of life, but when examined at 2.5 years of age, children from the fish oil group had a larger head circumference (mean difference: 0.5 cm (SD 0.2); *p* = 0.04) and a higher BMI (mean difference: 0.80 kg/m^2^ [SD 0.3]; *p* = 0.006) than those in the olive oil group [91]. Both body composition and head circumference at 2.5 years of age were also positively associated with the maternal erythrocyte DHA content. When followed up at 7 years of age, these effects were no longer evident, although higher diastolic blood pressure (DBP)(mean difference: 6.4 ± 2.1 mm Hg SD; *p* < 0.01) and mean arterial pressure (MAP) (mean difference: 6.6 ± 2.0 mm Hg SD; *p* < 0.01) was observed in boys from the fish oil group compared to the olive oil group [92]. No association was observed between maternal erythrocyte DHA and childhood blood pressure, suggesting that the observed difference may not have been caused by the intervention per se. Conversely, a population-based Dutch birth cohort study found that children at 12 years who were fed human milk with a relatively high content (above the median, i.e., ≥0.51 wt %) of LC n-3 PUFAs had a 4.8 mm Hg lower systolic blood pressure (SBP) (95% CI: −7.6 to −1.9) and a 2.5 mm Hg lower DBP (95% CI: −4.5 to −0.5) than children who were never breastfed [93]. Such effects are also seen in children that were born prematurely, such that in 13–16 year old children who were born prematurely, MAP was significantly lower in those assigned to banked breast milk than in those assigned to preterm formula (mean difference −4 × 1 mm Hg (95% CI: −6 × 6 to −1 × 6); *p* = 0.001) [94]. As some but not all studies have reported associations, further hypothesis-driven work is required to clarify these findings.

## 3. Conclusions & Future Research Priorities

Evidence from observational studies and randomized trials suggest a potential association between supplemental and dietary intake of LC marine n-3 PUFAs during pregnancy and some pregnancy and birth outcomes. With respect to maternal risk factors, the evidence generally does not support a role of maternal n-3 PUFA supplementation in altering the incidence of gestational diabetes, pregnancy-induced hypertension, or pre-eclampsia. In studies evaluating the duration of gestation, some discrepancies were observed, but most failed to detect a meaningful effect. It may be that benefit from n-3 PUFA supplementation is more pronounced in high-risk populations (i.e., women with a history of preterm birth or women with a low baseline n-3 PUFA intake), although further data are required in this area. Nonetheless, the cumulative evidence does not appear to be sufficient to recommend an n-3 PUFA supplementation for reducing the risk of preterm birth. Observational results concerning the effects of n-3 PUFA intake on maternal risk factors during pregnancy and offspring development are not conclusive, and intervention studies involving n-3 PUFA supplementation continue to yield equivocal evidence.

One mechanism for the proposed effects of n-3 PUFAs is their competition with n-6 PUFAs for incorporation into phospholipid membranes. However, many studies have not assessed biomarkers of n-3 PUFA intake, rather relying on the assessment of maternal fatty acid intake via dietary questionnaire which present a number of limitations. The assessment of specific n-6 or n-3 PUFA intake ratios in future studies will enable these possible metabolic interactions to be further detailed.

With regards to comparing efficacy of marine n-3 PUFA from dietary sources and fish oil supplements, the relative bioavailability of n-3 PUFAs from these difference sources and their structural forms (triglycerides, phospholipids, ethyl esters, free fatty acids) are of considerable importance. While most fish-derived EPA or DHA supplements are in the natural triglyceride form with similar bioavailability from food sources, it would be of interest to compare the n-3 PUFA uptake from marine n-3 PUFAs in different structural forms (e.g., Krill oil with n-3 PUFAs in phospholipids) and their influence on maternal risk factors. Furthermore, the inherent substitution of fish for a potentially less healthy meal (e.g., Red meat), may contribute to any benefits of fish consumption, but are not replicated by fish oil supplements. In the recent years, reports have emerged on oxidized fish oil being sold commercially. While the health implications of oxidized fish oil consumption remain unclear, it is important to note that previous trials may have been confounded by the use of oxidized oils. Future studies should report independent analyses of fish oils adopted in clinical trials and their oxidative status.

Finally, the discrepancies between studies, including trials, may be related to differences in study design, dosage, fatty acid interplay, or length of treatment. Further prospective double-blind studies and high quality observational dietary studies are needed to clarify the impact of long-chain marine n-3 PUFAs on these markers and risk factors for cardio-metabolic disease in the offspring.
